# Characteristics of percutaneous core biopsies adequate for next generation genomic sequencing

**DOI:** 10.1371/journal.pone.0189651

**Published:** 2017-12-27

**Authors:** Sharjeel H. Sabir, Savitri Krishnamurthy, Sanjay Gupta, Gordon B. Mills, Wei Wei, Andrea C. Cortes, Kenna R. Mills Shaw, Rajyalakshmi Luthra, Michael J. Wallace

**Affiliations:** 1 Department of Interventional Radiology, The University of Texas MD Anderson Cancer Center, Houston, Texas, United States of America; 2 Department of Pathology, The University of Texas MD Anderson Cancer Center, Houston, Texas, United States of America; 3 Department of System Biology, The University of Texas MD Anderson Cancer Center, Houston, Texas, United States of America; 4 Sheikh Khalifa Bin Zayed Al Nahyan Institute for Personalized Cancer Therapy, The University of Texas MD Anderson Cancer Center, Houston, Texas, United States of America; 5 Department of Biostatistics, The University of Texas MD Anderson Cancer Center, Houston, Texas, United States of America; 6 Department of Hematopathology, The University of Texas MD Anderson Cancer Center, Houston, Texas, United States of America; Rutgers University, UNITED STATES

## Abstract

**Purpose:**

Determine the characteristics of percutaneous core biopsies that are adequate for a next generation sequencing (NGS) genomic panel.

**Materials and methods:**

All patients undergoing percutaneous core biopsies in interventional radiology (IR) with samples evaluated for a 46-gene NGS panel during 1-year were included in this retrospective study. Patient and procedure variables were collected. An imaging-based likelihood of adequacy score incorporating targeting and sampling factors was assigned to each biopsied lesion. Univariate and multivariate logistic regression was performed.

**Results:**

153 patients were included (58.2% female, average age 59.5 years). The most common malignancy was lung cancer (40.5%), most common biopsied site was lung (36%), and average size of biopsied lesions was 3.8 cm (+/- 2.7). Adequacy for NGS was 69.9%. Univariate analysis showed higher likelihood of adequacy score (p = 0.004), primary malignancy type (p = 0.03), and absence of prior systemic therapy (p = 0.018) were associated with adequacy for NGS. Multivariate analysis showed higher adequacy for lesions with likelihood of adequacy scored 3 (high) versus lesions scored 1 (low) (OR, 7.82; p = 0.002). Melanoma lesions had higher adequacy for NGS versus breast cancer lesions (OR 9.5; p = 0.01). Absence of prior systemic therapy (OR, 6.1; p = 0.02) and systemic therapy </ = 3 months (OR 3.24; p = 0.01) compared to systemic therapy >3 months before biopsy yielded greater adequacy for NGS. Lesions <3 cm had greater adequacy for NGS than larger lesions (OR 2.72, p = 0.02).

**Conclusion:**

As targeted therapy becomes standard for more cancers, percutaneous biopsy specimens adequate for NGS genomic testing will be needed. An imaging-based likelihood of adequacy score assigned by IR physicians and other pre-procedure variables can help predict the likelihood of biopsy adequacy for NGS.

## Introduction

Image-guided percutaneous biopsy has traditionally been used in oncology to procure representative tissue from a radiologically detected lesion to determine histology. While this traditional role remains essential, acquisition of tissue to enable ancillary molecular testing is becoming increasingly important in today’s era of precision medicine.

Rapid advancements in genomic sequencing technology, including next generation sequencing (NGS), has allowed a growing number of genomic aberrations contributing to cancer pathogenesis to be discovered [1–5]. In parallel, pharmaceutical methods for targeting these mutations via small molecule inhibitors and antibodies are being developed [6–10]. When used in unselected patients, these agents demonstrate minimal activity; however, when used in patients whose tumors harbor the appropriate mutations, dramatic responses have been described [11–13]. Thus, implementation of precision medicine requires a high-quality tissue sample that can be tested for targetable genomic aberrations. Though surgical specimens are occasionally available, they are often collected at distant time points and thus may not represent the current state of the tumor. Therefore, percutaneous biopsy specimens are often used for molecular testing [14].

The increasing clinical and research demand for core biopsy specimens presents logistical challenges that must be considered when using these specimens for molecular testing. Given the small size of percutaneous biopsy specimens, NGS techniques with their lower DNA requirements hold great promise to provide broad molecular analysis [15], but compared to biopsy for histology, relatively tumor rich material with a significant number of tumor cells present is needed for current NGS testing [16]. An additional challenge due to tumor heterogeneity [17] is selection of the most appropriate lesion and the most appropriate component of that lesion. This problem can partially be addressed with advanced imaging techniques that can demonstrate the more cellular components of lesions [18].

This study was undertaken to determine the patient, lesion, and procedural technique variables associated with acquisition of percutaneous core biopsy specimens that are adequate for NGS.

## Materials and methods

This was a Health Insurance Portability and Accountability Act (HIPAA)-compliant, Institutional Review Board (IRB)-approved retrospective case series. This study was approved by the University of Texas MD Anderson Cancer Center IRB.

Informed consent/authorization to use and disclose protected health information was waived by the IRB because this was a retrospective case series that involved no diagnostic or therapeutic intervention, as well as no direct patient contact. Study staff were unable to obtain consent from study subjects because the patients were not specifically scheduled for follow up and may not have returned to MDACC; some were already deceased.

Identifiers (name, medical record number) were collected but were replaced by study numbers in the analytical file. Imaging/report dates were also collected as part of this study, in order to identify different imaging for the same patient. The key linking these numbers was retained in a locked file by the investigator. All study personnel completed training in methods for maintaining the confidentiality of health information. Electronic records were stored on password protected institution computers behind the institution firewall. Only the PI and research staff involved in the study had access to this data. Complete confidentiality was maintained during this retrospective evaluation, manuscript preparation and submission.

Data was kept on a secure computer at MD Anderson (password protected and located in a locked office). Paper records (data forms, list of patient names and unique identifiers, etc.) were kept in a locked file cabinet with access granted only to study investigators. Only key personnel were allowed to view data. Data from each patient was anonymized and assigned a unique identifier and stored where only the PI, Co-Chairs, Collaborators, and Research staff have access to the data.

All patients who had percutaneous core biopsies between 1/1/11 and 12/31/11 with specimens sent for NGS testing were included. Samples were sent for NGS when the patient’s oncologist requested sequencing of two or more genes in the tissue sample because of the lower DNA requirement with NGS (16). A total of 160 patients met inclusion criteria.

### Biopsy technique

When a biopsy request was received, an attending interventional radiology (IR) physician reviewed available diagnostic imaging and selected a target lesion. Written informed consent for biopsy was obtained. Anticoagulation and antiplatelet medications were held and coagulopathy was corrected before the procedure per consensus guidelines [19]. Biopsy procedures were performed by or under the supervision of attending IR physicians. The plans for NGS were not routinely available to IR physicians at the time of lesion selection or biopsy.

Most procedures were performed with moderate sedation, but occasionally general anesthesia was needed. After local anesthesia was obtained, a guiding cannula (13–19 gauge Chiba for soft tissue or 11–13 gauge Osteo-site for bone; Cook Inc.; Bloomington, IN) was inserted to the edge of the targeted lesion under image-guidance. Tissue specimens were obtained coaxially with fine needle aspirate (FNA) (22 g Chiba; Cook Inc.) and core biopsy (14–20 gauge Quikcore for soft tissue or Ackermann for bone; Cook Inc.) needles. The number of FNA and core specimens obtained, as well as the gauge of core biopsy needles used were determined by the attending IR physician performing the biopsy procedure.

### Biopsy specimen processing

FNA samples were smeared on glass slides and stained. On-site cytopathology assessment was available at the discretion of IR physician. The core samples were fixed in neutral buffered formalin, embedded in paraffin, and tissue sections were stained with hematoxylin and eosin (H&E).

When a request for genetic biomarker testing was received, a pathologist reviewed the H&E stained tissue sections of the core specimen and determined if there was at least 20% tumor cellularity in the specimen. If there was inadequate tumor cellularity, the sample was rejected for inadequate tissue. However, if there was >/ = 20% tumor cellularity and greater than 2 genetic biomarkers were requested, the sample was sent to our institution’s Clinical Laboratory Improvement Amendments (CLIA) certified Molecular Diagnostic Laboratory (MDL) for NGS.

### Next generation sequencing

The NGS platform used was Ion Torrent Personal Genome Machine (IT-PGM; Life Technologies, Carlsbad, CA) with the IT AmpliSeq cancer panel genomic library preparation protocol (Life Technologies) to test 46 genes. This specific NGS test will be referred to as cancer mutation screen 46 (CMS46). The amount of DNA was analyzed in the MDL and if found to be less than 10 ng (0.85 ng/μl), this was annotated as a failure due to inadequate DNA. Additional details of CMS46 are in Singh et al., [20].

### Data collection and analysis

The medical records of all patients were reviewed for the following parameters: age, gender, systemic therapy before biopsy, histologic diagnosis, fluorodeoxyglucose positron-emission tomography/computed tomography (FDG-PET/CT) </ = 6 months before biopsy, biopsy site, lesion size, primary tumor versus metastasis, imaging guidance, gauge of core biopsy, FNA acquisition, time between biopsy and NGS request, and adequacy for NGS. Complications were noted. The number and length of cores acquired were not routinely reported, but the routine practice during the study period was to obtain 2–3 FNA specimens followed by 3–4 core specimens, whenever feasible.

**Fig 1:**
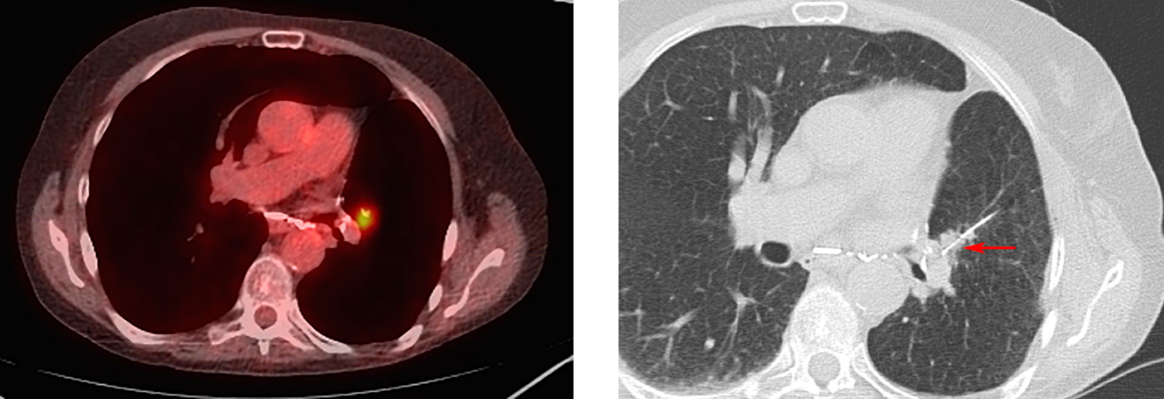
Example of likelihood of adequacy score 1 (low). **(A)** Fused PET/CT image showing a left lung nodule that though FDG avid is small, deep in emphysematous lung (better seen in b), and adjacent to the pulmonary artery. **(B)** Arrow points to needle in left lung nodule.

**Fig 2:**
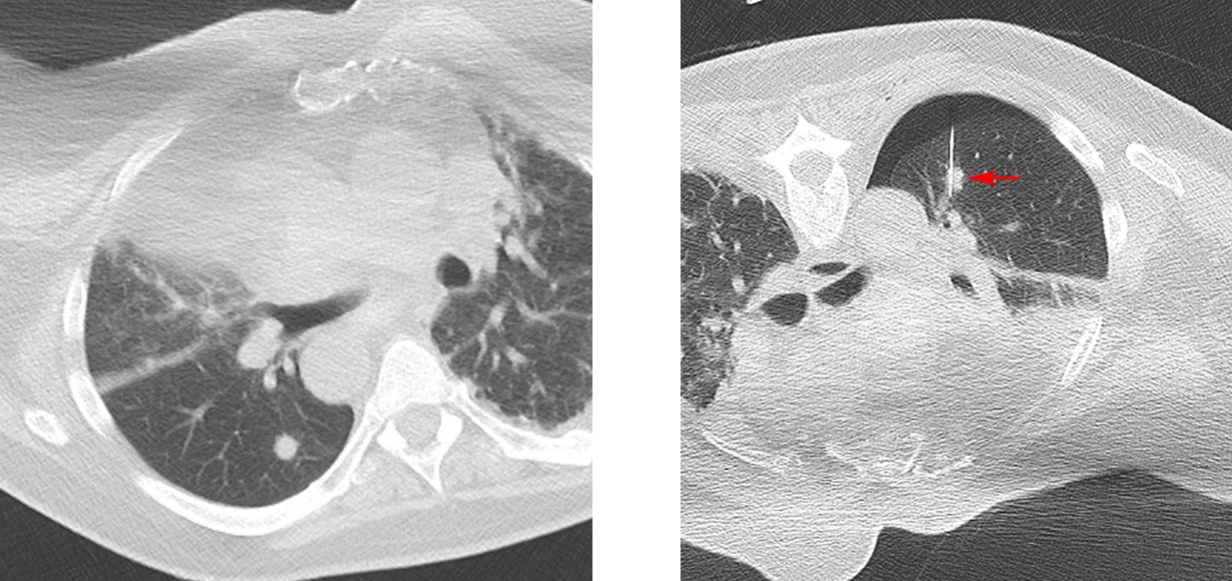
Example of likelihood of adequacy score 2 (equivocal). **(A)** Chest CT showing right lung nodule that is small and in location (behind rib) requiring angled approach **(B)** Arrow points to needle in right lung nodule. Small pneumothorax is noted.

**Fig 3:**
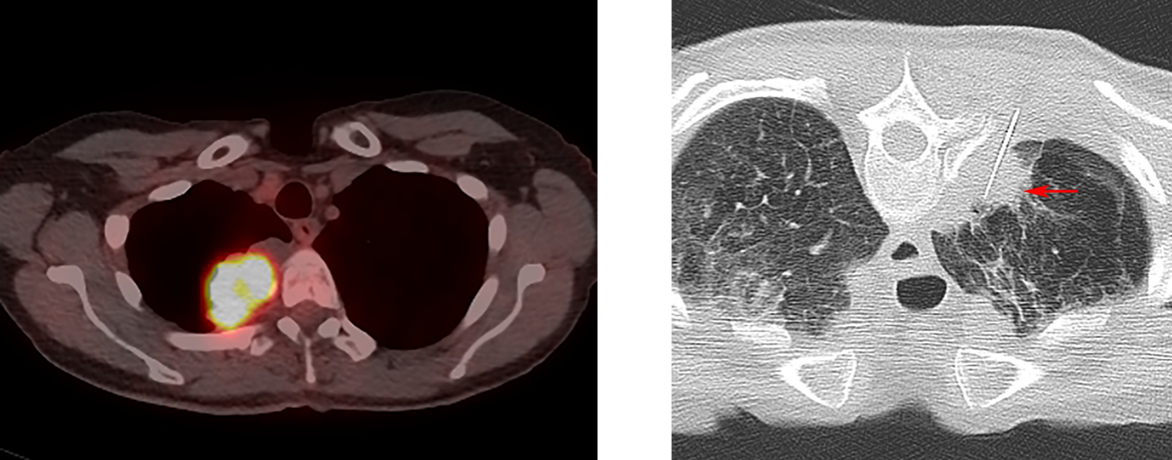
Example of likelihood of adequacy score 3 (high). **(A)** PET/CT showing right lung lesion that is large and FDG avid. Also, there is no aerated lung in the planned biopsy path. **(B)** Arrow points to needle in right lung mass.

Two attending IRs with 1 (SHS) and 19 (MJW) years of experience reviewed pre-procedure imaging while blinded to the results of NGS testing. A likelihood of adequacy score was assigned based on lesion imaging characteristics that reduced the likelihood of adequate lesion targeting and sampling (Table 1). The scale used ranged from score 1 representing low likelihood of adequacy for NGS assigned when three or more equally weighted problematic characteristics were present or a highly problematic factor (i.e. any of the listed targeting or sampling factors manifesting in an extreme form such as a lesion completely surrounded by high risk structures or a completely sclerotic lesion) was present (Fig 1), score 2 representing equivocal likelihood of adequacy for NGS when two problematic characteristics were present (Fig 2), to score 3 representing high likelihood of adequacy for NGS when no more than one problematic characteristic was present (Fig 3).

**Table 1:** Problematic lesion imaging characteristics affecting likelihood of adequacy score^a^.

Targeting Factors	Sampling Factors
Small (<2cm) size	Sclerosis
Proximity to high risk structures^b^	Necrosis^f^
Unfavorable surrounding tissue^c^	
Highly angled approach^d^	
Location susceptible to motion^e^	

a: Likelihood of adequacy score: 1 (low) when ≥ 3 factors were present or a highly problematic factor (i.e. any of the listed targeting or sampling factors manifesting in an extreme form such as a lesion completely surrounded by high risk structures or a completely sclerotic lesion) was present, 2 (equivocal) when 2 factors present, and 3 (high) when ≤ 1 factor present

b: High risk structure is defined as any structure that must not be traversed by the biopsy needle, e.g. heart, aorta, colon, major nerves, etc

c: Unfavorable surrounding tissue is defined as tissue that is at risk for complications when traversed by the biopsy guide needle such as emphysematous lung or might be challenging to traverse such as bone.

d: A highly angled approach that requires a significantly out of plane trajectory can make it difficult to confidently reach the target lesion, especially with CT guidance

e: Some locations are associated with significant motion whether respiratory motion such as peridiaphragmatic lung or cardiac motion such as pericardial lesions, which makes accurate targeting challenging

f: Necrosis is suggested by lack of contrast enhancement and/or FDG uptake

A biopsy yielding material for histological assessment was considered technically successful. A biopsy yielding sufficient genetic material to allow the CMS46 panel to be performed was considered adequate for NGS. Descriptive statistics were collected. Univariate logistic regression analyses were performed to determine influences on biopsy adequacy for NGS. Using backwards elimination starting from all variables described above a multivariate logistic regression model (SAS 9.2; SAS Institute Inc., Cary, NC) was fitted to the data to investigate the presence of higher dimension associations. A p value <0.05 was considered statistically significant.

## Results

**Fig 4:**
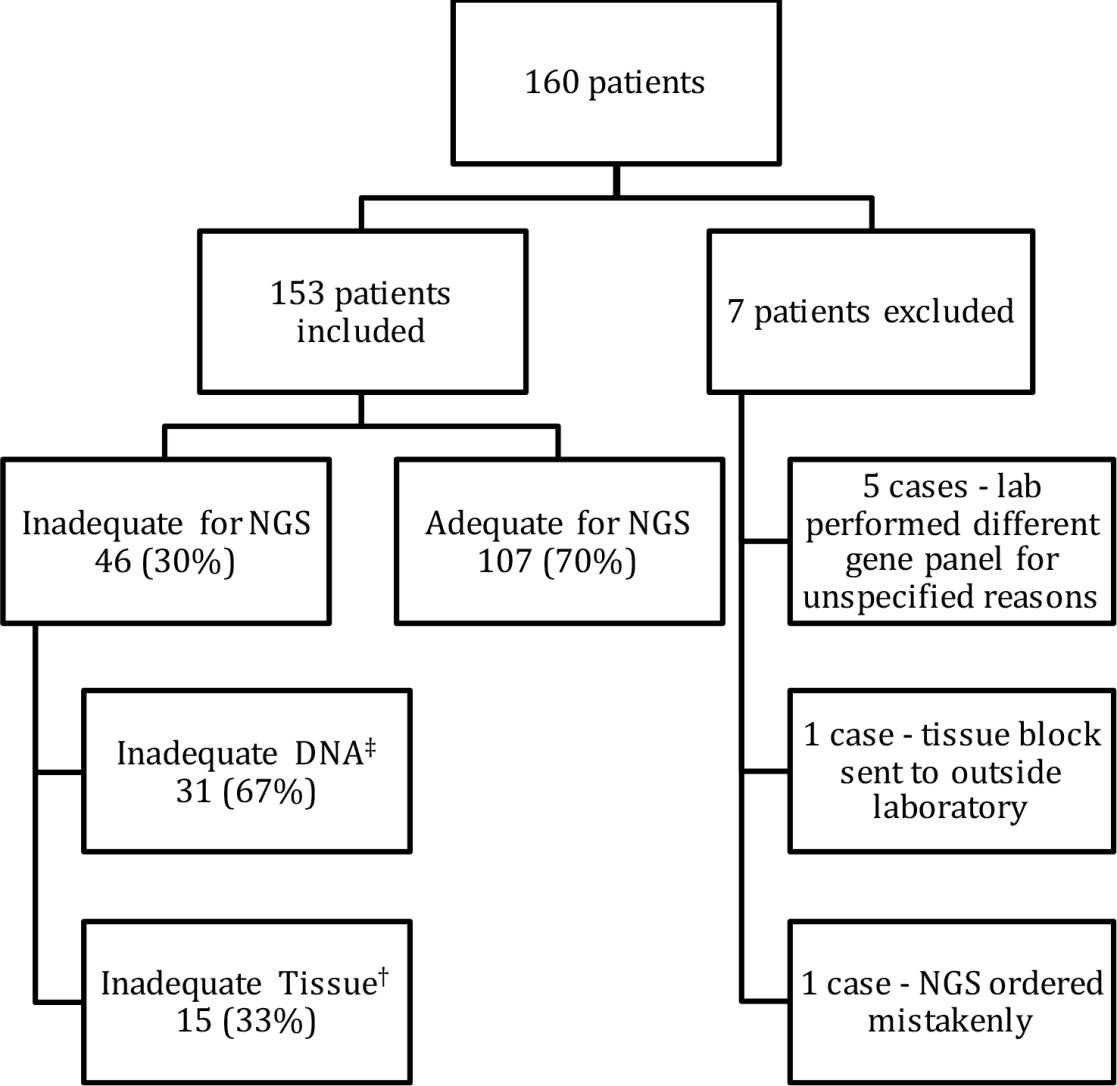
Patient inclusion, overall adequacy for NGS, and reason inadequate for NGS. Footnote: NGS: Next generation sequencing. ^a^ DNA quantity < 10 ng. ^b^ Less than 20% tumor cellularity due to necrosis, fibrosis and quantity of tissue available for analysis.

All 160 patients had technically successful percutaneous biopsies adequate for making a histologic diagnosis. 7 patients were excluded from the analysis of adequacy for NGS because CMS46 testing was not performed by the MDL (Fig 4).

Of the included 153 patients, 89/153 (58.2%) patients were female. The average age of patients was 59.5 years (+/- 12.2). The average size of biopsied lesions was 3.8 cm (+/- 2.7). The most common malignancy in the biopsy specimens was lung cancer (40.5%). The most common sites biopsied were lung (36%) and liver (27.5%). No prior systemic therapy had been given in 25/153 (16.3%) patients. CT guidance was most commonly used (60.1%). 20g core needles were used in the majority of cases (88.2%). PET scan was performed within 6 months before the biopsy in 82/153 (53.6%) patients. The average time between biopsy and MDL request was 68 days (+/- 122). Additional variables are noted in Tables 2 and 3.

**Table 2:** Patient and technique variables with adequacy for NGS of biopsy specimens.

Characteristic	Adequate for NGS n (%)	Inadequate for NGS n (%)	N (%)
**Total Included Patients**			153 (100)
**Gender**			
**Female**	57 (64)	32 (36)	89 (58.2)
**Male**	50 (78.1)	14 (21.9)	64 (41.8)
**Primary Malignancy**			
**Lung**	48 (77.4)	14 (22.6)	62 (40.5)
**Breast**	25 (56.8)	19 (43.2)	44 (28.8)
**Melanoma**	15 (88.2)	2 (11.8)	17 (11.1)
**Other**	19 (63.3)	11 (36.7)	30 (19.6)
**Histology**			
**Carcinoma**	86 (67.2)	42 (32.8)	128 (83.7)
**Melanoma**	16 (88.9)	2 (11.1)	18 (11.8)
**Other**	5 (71.4)	2 (28.56)	7 (4.5)
**Biopsy Site**			
**Lung**	42 (76.4)	13 (23.6)	55 (36)
**Liver**	31 (73.8)	11 (26.2)	42 (27.5)
**Bone**	7 (46.7)	8 (53.3)	15 (9.8)
**Lymph Node**	11 (78.6)	3 (21.4)	14 (9.1)
**Other**	16 (59.3)	11 (40.7)	27 (17.6)
**Metastasis vs Primary**			
**Metastasis**	87 (69.1)	39 (30.9)	126 (82.4)
**Primary**	20 (74.1)	7 (25.9)	27 (17.6)
**Lesion Diameter <3 cm**			
**Yes**	53 (75.7)	17 (24.3)	70 (45.8)
**No**	54 (65.1)	29 (34.9)	83 (54.2)
**Systemic Therapy before Biopsy**			
**Never treated**	22 (88)	3 (12)	25 (16.3)
**</ = 3 months**	60 (71.4)	24 (28.6)	84 (54.9)
**> 3 months**	25 (56.8)	19 (43.2)	44 (28.8)
**Guidance method**			
**Computed Tomography**	63 (68.5)	29 (31.5)	92 (60.1)
**Ultrasound**	43 (72.9)	16 (27.1)	59 (38.6)
**Magnetic Resonance Imaging**	1 (50)	1 (50)	2 (1.3)
**CNB gauge**			
**20G**	97 (71.8)	38 (28.2)	135 (88.2)
**18G**	7 (77.8)	2 (22.2)	9 (5.9)
**Other (11–16 g)**	3 (33.3)	6 (66.7)	9 (5.9)
**Concurrent FNA**			
**Yes**	88 (69.8)	38 (30.2)	126 (82.4)
**No**	19 (70.4)	8 (29.6)	27 (17.6)
**Likelihood of Adequacy for NGS Score**			
**1 (low)**	5 (33.3)	10 (66.7)	15 (9.8)
**2 (equivocal)**	19 (65.5)	10 (34.5)	29 (19)
**3 (high)**	83 (76.2)	26 (23.8)	109 (71.2)
**PET within 6 months of Biopsy**			
**Yes**	60 (73.2)	22 (26.8)	82 (53.6)
**No**	47 (66.2)	24 (33.8)	71 (46.4)

Note.—NGS: Next generational sequencing, PET: Positron emission tomography

**Table 3:** Patient age and days between biopsy and NGS request with adequacy for NGS of biopsy specimens.

Characteristic	N	Mean	SD	Min	Median	Max
Age						
Adequate for NGS	107	59.6	12.4	21	59	93
Inadequate for NGS	46	59.1	11.7	23	61	84
All	153	59.5	12.2	21	60	93
Days between biopsy and NGS request						
Adequate for NGS	107	60	116.9	0	9	701
Inadequate for NGS	46	87.6	133.4	0	33	710
All	153	68.3	122.3	0	13	710

Note.—NGS: Next generation sequencing, SD: Standard deviation

**Fig 5:**
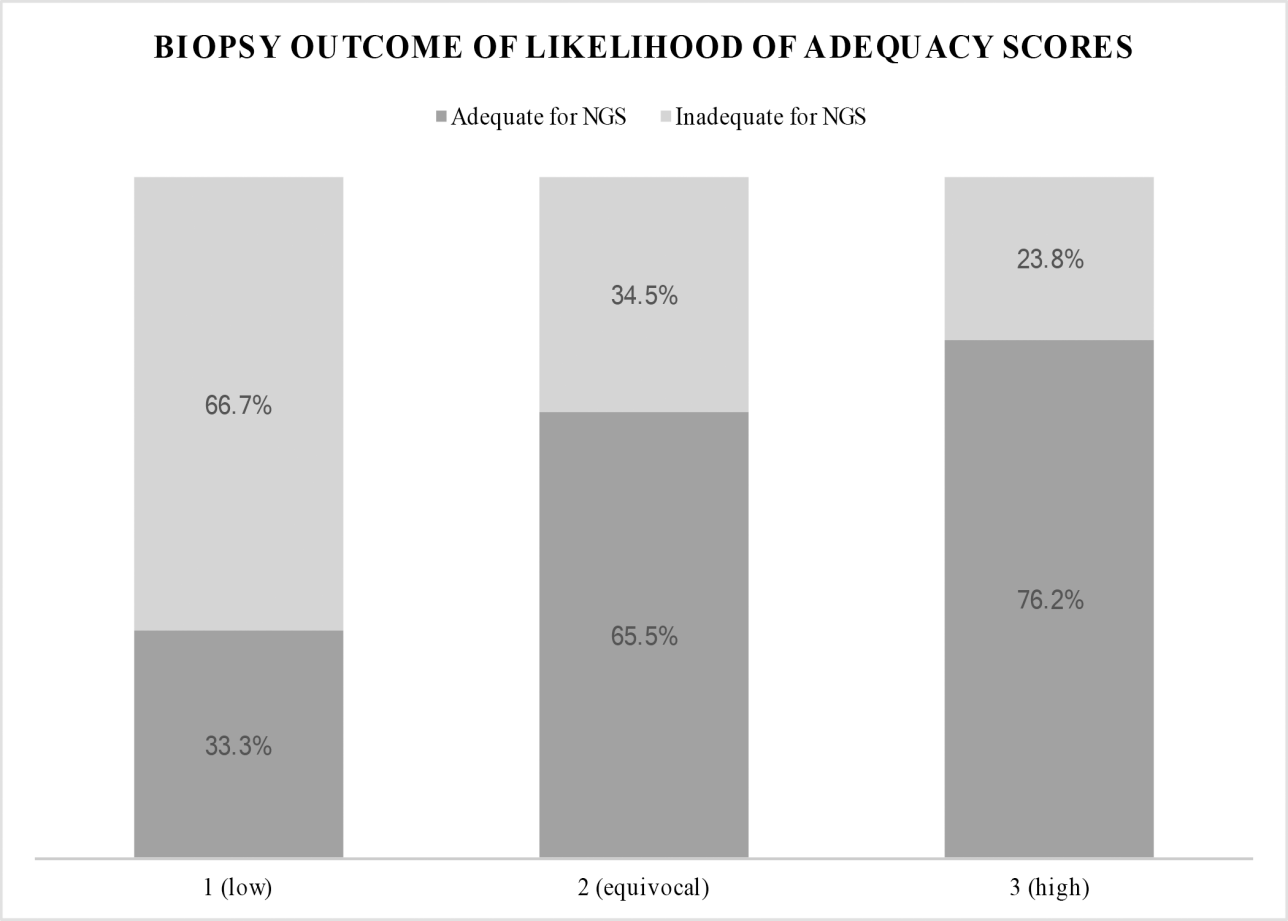
Biopsy outcome of likelihood of adequacy scores. Footnote: NGS: Next Generation Sequencing.

The adequacy for NGS of each likelihood of adequacy score was 33.3% for a score of 1, 65.5% for a score of 2, and 76.2% for a score of 3 (Fig 5). A score of 3 was the most commonly assigned score. The kappa coefficient for the likelihood of adequacy score assigned by two reviewers (SHS and MJW) based on pre-procedure imaging review blinded to results of NGS testing was 0.53 (95% CI: 0.40–0.65) representing moderate agreement. In the subsequent univariate and multivariate analysis reported in this study, the likelihood of adequacy score assigned by SHS was used. The results of the univariate and multivariate analysis with the score assigned by MJW were similar.

The overall adequacy for NGS was 107/153 (69.9%) with the association between adequacy for NGS and individual variables shown in Table 4. The cause of the inadequacy was inadequate tissue as designated by the reviewing pathologist in 15/46 (32.6%) cases while inadequate DNA as determined by quantification of DNA amount in our MDL was the cause in 31/46 (67.4%) cases (Fig 4). Given the small numbers for the subtypes of failure, logistic regression was limited to overall adequacy for NGS.

**Table 4:** Univariate logistic regression model of characteristics associated with adequacy for NGS of biopsy specimens.

Characteristic	Odds Ratio	95% LCL	95% UCL	Pairwise p-value	Overall p-value
**Age**					
1 year increase	1.004	0.975	1.033	0.804	
**Gender**					
Male vs. Female	2.005	0.977	4.273	0.063	
**Primary Tumor**					**0.030**
Other vs. Breast	1.313	0.510	3.464	0.576	
Melanoma vs. Breast	5.700	1.383	38.989	0.032	
Lung vs. Breast	2.606	1.131	6.153	0.026	
**Histology**					0.125
Other vs. Carcinoma	1.221	0.252	8.767	0.816	
Melanoma vs. Carcinoma	3.907	1.046	25.431	0.078	
**Biopsy site**					0.143
Other vs. Bone	1.662	0.466	6.096	0.434	
Lung vs. Bone	3.692	1.127	12.532	0.031	
Lymph node vs. Bone	4.191	0.880	24.624	0.085	
Liver vs. Bone	3.221	0.951	11.345	0.061	
Primary or Metastasis					
Primary vs. Metastasis	1.281	0.519	3.489	0.606	
**Tumor size**					0.150
<3cm vs. > = 3cm	1.674	0.831	3.450	0.154	
**Systemic Therapy Naïve**					
Yes vs. No	3.710	1.200	16.281	0.042	
**Systemic Therapy before Biopsy**					**0.018**
</ = 3 vs. > 3 months	1.900	0.886	4.086	0.099	
Never vs. > 3 months	5.573	1.624	26.022	0.012	
**Guidance Modality**					0.709
US vs. CT	1.237	0.605	2.588	0.564	
MRI vs. CT	0.460	0.018	11.901	0.588	
**Core Needle Gauge**					0.060
Other vs. 18 G	0.143	0.014	1.026	0.069	
20 G vs. 18 G	0.729	0.106	3.180	0.702	
**PET within 6 months**					
Yes vs. No	1.393	0.696	2.800	0.349	
**Likelihood of Adequacy for NGS Score**					** 0.004**
3 vs. 1	6.385	2.076	22.102	0.002	
2 vs. 1	3.800	1.055	15.252	0.047	
**Days from biopsy to NGS**					
1 day increase	0.998	0.996	1.001	0.213	

Odds ratio higher than 1 means higher probability of adequacy for NGS. Overall p-values are for factors with 3 or more levels. NGS: Next generation sequencing, FNA = Fine Needle Aspiration Biopsy, FDG: Fluorodeoxyglucose (^18^F), PET: Positron emission tomography

Univariate logistic regression showed that the statistically significant variables were primary malignancy (p = 0.03) with pairwise comparison showing higher odds ratio of adequacy for NGS of melanoma vs. breast (p = 0.032) and also lung vs. breast (p = 0.026). Additionally, there was increased likelihood of adequacy in systemic therapy naïve patients vs. all patients (p = 0.042) and even greater odds of adequacy vs. patients who received systemic therapy >3 months before biopsy (p = 0.012). Finally, the IR assigned likelihood of adequacy score was statistically significant (p = 0.0004) including pairwise comparison of scores of 3 vs. 1 (p = 0.002) and 2 vs. 1 (p = 0.047) (Table 4).

In multivariate logistic regression analysis, several factors were found to be statistically significant. When comparing the various primary malignancies, breast cancer lesions had significantly worse yield compared to melanoma lesions (p = 0.004). Patients who had never received systemic therapy (p = 0.01) or had systemic therapy within 3 months of biopsy (p = 0.02) had better likelihood of adequacy than those who received systemic therapy greater than 3 months before the biopsy. A likelihood score of 3 vs. 1 was associated with higher odds of adequacy for NGS (p = 0.002). A factor that became apparent as statistically significant in multivariate analysis was lesions <3 cm in diameter had a better likelihood of adequacy for NGS compared to lesions >/ = 3 cm (Table 5).

**Table 5:** Multivariate logistic regression model predicting adequacy for NGS of biopsy specimens.

Characteristic	Odds Ratio	95% LCL	95% UCL	p-value
**Primary Tumor**				
Other vs. Breast	1.33	0.47	3.84	0.60
Melanoma vs. Breast	9.50	1.84	76.88	**0.01**
Lung vs. Breast	2.20	0.83	5.99	0.12
**Tumor Size**				
<3 vs. >/ = 3 cm	2.72	1.18	6.66	**0.02**
**Likelihood of Adequacy Score**				
3 vs. 1	7.82	2.19	32.11	**0.002**
2 vs. 1	2.96	0.71	13.61	0.15
**Prior Systemic Therapy**				
</ = 3 vs. > 3 Months	3.24	1.33	8.23	**0.01**
Never Treated vs. > 3 Months	6.10	1.50	32.40	**0.02**

Note.—Odds ratio higher than 1 means higher probability of adequacy for NGS. NGS: Next generation sequencing, LCL: lower confidence limit, UCL: Upper confidence limit

Procedural complications were noted in 10/153 (6.5%) procedures. All complications were pneumothoraces related to lung biopsies with 6 patients requiring chest tubes and the rest managed conservatively.

## Discussion

Precision medicine holds the promise of improved outcomes by targeting patient specific molecular aberrations. Percutaneous core biopsy is a central method for acquiring material for molecular diagnosis; however, there are logistical challenges to using these specimens as evidenced by the wide spectrum of genetic sequencing adequacy of biopsy samples ranging from 39% to 95% in trials using sequencing for treatment selection with the higher yields generally found with surgical specimens, fresh frozen rather than formalin fixed specimens, or testing for fewer genes [21–30]. Though our reported 69.9% adequacy for NGS in a cohort of patients with histologically diagnostic biopsies is well within the reported range, the reasons for lower adequacy for NGS compared to histological diagnosis requires evaluation [14]. Our study illustrates several factors associated with the outcome of percutaneous core biopsy specimens being used for NGS that could help refine the process.

A novel imaging-based likelihood of adequacy score assigned by an IR physician was found to be significantly associated with adequacy of percutaneous biopsy for NGS on both univariate and multivariate analysis. A likelihood score of 3 (high likelihood) had an odds ratio of 7.82 compared to lesions scored as 1 (low likelihood) on multivariate analysis. There was moderate agreement (kappa 0.53) between two raters. This level of agreement is comparable to that reported for other clinically used assessment scores [31]. In addition, there was statistically significant correlation between the score assigned and likelihood of adequacy for NGS on multivariate analysis for both raters, thus even though for any given patient there might be slight disagreement between the score assigned by the raters, overall the likelihood score assigned by attending interventional radiologists, whether early in their careers or with significant experience, correlated with adequacy for NGS.

The type of cancer being biopsied affected the yield for NGS, a finding partially supported by a recent study that showed histological subtype influenced adequacy of biopsy [30] but other recent studies have shown no association [24, 28]. On multivariate analysis, melanoma lesions had higher odds of adequacy for NGS than breast cancer lesions (OR 9.5). This result might be attributable to differing tumor microenvironments, specifically the potentially increased amount of fibrosis present in breast cancer lesions versus melanoma lesions [32].

Unlike prior studies that showed no associated between lesion size and adequacy of biopsy [21, 28, 30], in our study lesions <3 cm in maximal diameter had higher odds (OR 2.72) of adequacy for NGS than larger lesions on multivariate analysis. This result was unexpected because of the ease with which larger lesions are biopsied. However, as lesions enlarge, they usually outgrow their blood supply and tend to develop necrosis [33]. Our practice is to target the periphery of large lesions to avoid sampling the potentially necrotic core. We target FDG avid areas for biopsy, if FDG-PET imaging is available, given the association between FDG avidity and tumor cellularity [18]. Despite these efforts, lesions >/ = 3 cm in diameter had lower likelihood of adequacy for NGS, perhaps due to necrosis in areas beyond the center of the tumor, which is supported by the study by Tacher et al., showing that there is no difference in biopsy adequacy between specimens acquired from the center or the periphery of a lesion [28]. Additionally, we showed, as in prior work [21], that availability of PET imaging does not seem to help improve adequacy of biopsy.

Multivariate analysis showed patients with no prior systemic therapy (OR 6.1) or systemic therapy </ = 3 months before biopsy (OR 3.24) had higher adequacy for NGS than patients who received systemic therapy >3 months before biopsy. This result might be related to the increasing proportion of fibrosis versus viable tumor developing over time in lesion exposed to systemic therapy >3 months before biopsy [34, 35]. In contrast, lesions not exposed to prior systemic therapy or systemic therapy closer to time of biopsy might be less likely to have developed as much fibrosis. This finding contrasts with prior work showing that ongoing chemotherapy at the time of biopsy reduces yield [30] or has no effect on biopsy yield [28]. The difference might be attributable to the different tumor types included in those studies compared to ours. For instance, our study included a significant number of melanoma lesions, while the studies by Tacher et al., and Desportes et al., had few if any melanoma lesions included.

The amount and quality of tissue acquired with a percutaneous biopsy are only the initial challenges to successful NGS with biopsy material. Additional challenges are related to stabilization, storage, selection, dissection, molecular extraction, and sequencing of specimens along with overall coordination of the process [14, 15, 36]. For instance, in our study there were 7 cases that had to be excluded because NGS was not performed for reasons other than tissue amount and quality. In addition, over time, the amount of DNA required for NGS continues to change; for example the CMS46 panel required 10 ng of DNA during our study period, but our pathology group eventually refined the sequencing protocol and were able to perform the test with <10 ng of DNA [24]. Thus, NGS testing needs thorough coordination between requesting physicians, interventional radiologists, and pathologists.

The limitations of our study include selection bias that is inherent in any retrospective study. We did not compare the NGS yield in patients without histologically diagnostic biopsies, which would potentially have reduced the yield of percutaneous biopsy for NGS. The NGS likelihood score is somewhat subjective and additional development is needed to allow reproducible validation. We were not able to collect the number of cores taken during the biopsy procedure, which could be a confounding variable and will have to be addressed in subsequent studies. Although our results seem biologically plausible, the issue of heterogeneous population and multiple comparisons is a concern and our results must be substantiated in additional patient cohorts.

In conclusion, NGS testing of percutaneous biopsy specimens is becoming more prevalent both in trial setting as well as in routine clinical care. Our study demonstrated various factors contributing to the adequacy of percutaneous core biopsies for NGS testing, but ultimately optimal outcomes require clear communication between requesting physicians, interventional radiologists, and pathologists so biopsies are planned, performed, and managed in a coordinated fashion.
